# Administration of Nitrous Oxide by Medical Assistants for Painful Procedures in Outpatient Pediatric Settings

**DOI:** 10.3390/children11091091

**Published:** 2024-09-06

**Authors:** Colette Balice-Bourgois, Luciano Anselmi, Barbara Schild, Maya Zumstein-Shaha, Mario Mendoza-Sagaon

**Affiliations:** 1 Department of Pediatric, Institute of Pediatrics of Southern Switzerland, Ente Ospedaliero Cantonale, 6500 Bellinzona, Switzerland; 2 Department of Anesthesia, Institute of Pediatrics of Southern Switzerland, Ente Ospedaliero Cantonale, 6500 Bellinzona, Switzerland; luciano.anselmi@eoc.ch (L.A.); barbara.schild@eoc.ch (B.S.); 3 Department of Health Professions, Applied Research & Development in Nursing, Bern University of Applied Sciences, 3010 Bern, Switzerland; maya.zumsteinshaha@bfh.ch; 4 School of Nursing Science, Faculty of Health, Department of Nursing Science, Witten/Herdecke University, 58455 Witten, Germany; 5 Department of Pediatric Surgery, Institute of Pediatrics of Southern Switzerland, Ente Ospedaliero Cantonale, 6500 Bellinzona, Switzerland; mario.mendozasagaon@eoc.ch

**Keywords:** procedural pain, nitrous oxide, medical assistant, pediatric setting, ambulatory care, pain management

## Abstract

Background: Managing pain and distress in children experiencing procedural pain is a priority in pediatric care. Nitrous oxide (pre-mixed formulation of 50% nitrous oxide to 50% oxygen) is widely used to alleviate anxiety or pain during care procedures in various medical domains. This pharmacological intervention is safe to administer to children. Administration by non-anesthesiologist personnel is widespread throughout the world, though it is almost exclusively performed by doctors and nurses. The purpose of this study is to describe the experience of nitrous oxide (N_2_O) use by medical assistants and safe handling during the performance of painful procedures. Methods: A retrospective study was conducted in a pediatric outpatient unit of a regional hospital, including medical and surgical consultations. Results: Nitrous oxide was administered by medical assistants to 324 children aged 3 to 19 years. No respiratory and/or cardiovascular problems were noted. Most patients were calm and relaxed. Discussion: The use of N_2_O for minor procedures in children in a pediatric outpatient unit improved pain management through administration by medical assistants with a high level of satisfaction from patients, parents and health professionals. Conclusions: This study suggests that the use of nitrous oxide by trained medical assistants can be safe, feasible and effective within the specific context of this study.

## 1. Introduction

Painful procedures are frequently encountered in pediatrics. Managing pain and distress in children experiencing procedural pain is a priority when providing care, regardless of whether the child is hospitalized or in ambulatory care. Different approaches or treatments to minimize pain and distress are available [1,2]. One of these is the use of nitrous oxide in a pre-mixed formulation of 50% nitrous oxide to 50% oxygen (or MEOPA—Mélange Equimolaire Oxygene Protoxyde d’Azote, or the commercial versions Entonox^®^ and Antasol^®^) [3]. In Switzerland, the mixture administered is called KALINOX^®^, which is available in various sizes and is authorized by Swissmedic (authorization number 57799) for use in pediatric and adult populations for emergency treatment, short-term painful operations, dental care and obstetrics [4].

Nitrous oxide is widely used to alleviate anxiety or pain during care procedures in various medical domains, such as pediatrics, oncology, the emergency room, dentistry and home care [5,6,7]. The use of nitrous oxide in concentrations up to 50% with oxygen during pediatric procedures is effective and safe to administer to children to manage pain and anxiety [1,8]. The use of nitrous oxide outside the operating room is accepted and well documented [6,7,9,10,11,12,13,14]. Generally, nitrous oxide is well tolerated, as most of the adverse observed effects are minor (e.g., vomiting). Major adverse events are very rare and are usually encountered when patients have received psychotropic drugs (benzodiazepine or opioid) in addition to nitrous oxide or when the inhalation time exceeds 30 min [5,7,12,15,16,17].

Nitrous oxide has both analgesic and anxiolytic effects. It has a quick onset of action, as well as the advantage of rapid recovery after discontinuation. Thus, it provides sedation and analgesia with spontaneous respiration, and it has a minimal effect on the hemodynamic status and protective airway reflexes of the patient [6,9,13]. In a randomized controlled trial, nitrous oxide inhalation was shown to be more effective in reducing distress and had fewer adverse effects and shorter recovery times than midazolam [18]. Another advantage of using nitrous oxide is that it does not require mandatory fasting [3,4,14,19]. This is particularly beneficial for pediatric outpatient procedures and emergency departments, where the ability to administer sedation quickly and safely without the need for the patient to fast first can improve care and reduce patient pain and distress.

Administration by non-anesthesiologist personnel trained in the use of nitrous oxide is widespread throughout the world [7,9,20,21]. Among the group of non-medical health professionals are midwives, physiotherapists, paramedics, medical imaging technicians, and others. All of these professionals have received specific training in administering nitrous oxide [3,9,10,22]. Nitrous oxide has also been used in outpatient and home settings for various painful procedures in children and adults [11]. In conclusion, nitrous oxide can be used by any trained health professional and not only by a physician or a nurse provided it has been prescribed by a physician [4,23].

Adoption of non-medical professionals administering nitrous oxide has grown in Switzerland in recent years. Nevertheless, nurses still predominantly administer nitrous oxide today. The possibility of nitrous oxide being administered by medical assistants has been considered by anesthesiologists and pediatricians/surgeons. It is believed that involvement of medical assistants will improve pain control and anxiety management in pediatric consultations.

In Switzerland, pediatric general practices are run by pediatricians (physicians) and medical assistants. Such an assistant is a person who manages the different tasks related to a medical or surgical consultation such as identifying the electronic record, performing venipuncture, dressing burn wounds, assisting in minor surgical procedures, handling administrative issues, etc. (a medical assistant is not an intern or a resident). Rarely, other healthcare providers such as nurses work in general or pediatric practice or similar settings. Therefore, the absence of nurses in medical consultation rooms/general practices or in outpatient clinics allowed for studying the use of nitrous oxide by medical assistants and ensured equitable access to procedural pain relief [22].

The purpose of this study was to describe the experience of medical assistants handling nitrous oxide and the way these assistants use nitrous oxide in painful procedures in pediatric outpatient’s clinics or similar settings.

## 2. Materials and Methods

### 2.1. Study Setting

This retrospective study was conducted in the pediatric outpatient clinic of a regional hospital in Southern Switzerland with internal medicine and surgical consultations. This clinic carries out 22,000 such consultations per year. The study is based on anonymized patient data from 2019 to 2021 and, therefore, did not require the approval of the respective Ethics Committee as it was considered quality assurance work (request ethics committee 2021-00509).

### 2.2. Implementation Strategy of Nitrous Oxide in Pediatric Outpatient Clinics by Medical Assistants

In order to safely implement the administration of nitrous oxide for painful procedures, a protocol including indications, contraindications, equipment, administration, potential problems and recommendations, as well as a comprehensive specific training program, was developed:

- Theoretical training given by an anesthetist physician with content on product knowledge, administration methods, knowledge of indications and contraindications and monitoring;
- Practical training by a specialist nurse in practical workshops;
- Performance of at least eight administrations of nitrous oxide under nurse supervision (one-on-one guidance), with the completion of a skills assessment grid upon each administration of nitrous oxide.

### 2.3. Method of Delivery

Nitrous oxide was only used in a room specifically equipped for small procedures and minor surgical interventions, where appropriate equipment was available in case of adverse events. The use of the ready-to-use mixture (50% N_2_O–50% O_2_) is compulsory for safety reasons as it prevents the need to titrate the N_2_O for the patient [4,9,11]. Nitrous oxide was used in children between 3 and 19 years of age who needed to undergo painful procedures. The only exclusion criterion was being under 3 years of age. All procedures with nitrous oxide were performed by two medical assistants (one assistant administered the nitrous oxide and the other medical assistant assisted and helped the physician performing the procedure. During painful procedures with nitrous oxide, the medical assistant administering nitrous oxide needed to complete a monitoring form. In this form, the medical prescription of the painful procedure was stated including any other medication (local anesthesia or paracetamol/NSAID) and signed by the respective physician. The medical assistant also needed to note monitoring results (i.e., O_2_ saturation, heart rate, respiratory rate and pain) as well as any side effects on this form. Before the painful procedure, a form with the patient’s medical background had to be completed and signed by the parents. On this form, it was possible to mention cases of skin breakdown so that local anesthetics (such as EMLA, etc.) could be used.

For the first nitrous oxide inhalation, it was necessary to prepare and train the child in the presence of the parents. The child was able to choose a scent (chocolate, strawberry or vanilla) to be added to the mask. Subsequently, the mix had to be inhaled for three minutes before the procedure started. A 50% nitrous oxide and 50% oxygen premix (Kalinox^®^) was used and administered in continuous flow. The child was monitored throughout the procedure with a pulse oximeter, while being reassured and distracted by the second medical assistant. The duration of inhalation was not to exceed 20 min. At the end of the procedures, the medical assistant administering the nitrous oxide assessed pain and ensured that patients had a rapid recovery.

### 2.4. Data Collection and Statistical Analysis

For the purpose of the study, the following data from the monitoring forms were collected and analyzed:

- Demographic data;
- Type of procedure;
- Pain;
- Inhalation time of nitrous oxide;
- Side effects;
- Oxygen saturation and heart rate (before, during and after the procedure);
- Other analgesics;
- Other topical or local anesthetic.

Descriptive statistics (frequency, mean, and standard deviation) were used to summarize the characteristics of the study sample.

## 3. Results

A total of N = 324 painful procedures, i.e., pediatric surgery, were performed with nitrous oxide, of which n = 204 involved male and n = 120 female patients with an age range of 3 to 19 years (mean 10.4 years, ±3.7) (Table 1 and Table 2).

The inhalation time ranged from 4 to 50 min with an average of 9.6 min. In more than half of the cases, the administration of nitrous oxide was combined with one or two other analgesics and/or local anesthetics to improve pain management: EMLA^®^ cream (n = 101), local injection of xylocaine (n = 92), paracetamol (n = 4), NSAIDs (n = 18) and nasal fentanyl (n = 1) (Table 3).

All oxygen saturation values ranged between 93% and 100% during inhalation (mean value 99.5%). The heart rate varied between 46 and 130 beats with an average of 86 beats.

The Faces Pain Scale-Revised [24] was only used with two children (1/10 and 7/10). Overall, pain was rated as absent or moderate in 321 cases and severe in 2 cases. Most patients were reported being calm and relaxed. The use of nitrous oxide was considered very successful by the entire healthcare team as well as the parents.

In total, six adverse reactions were recorded in six children: vomiting (n = 1), agitation (n = 2), severe pain (n = 2) and anaphylactic reaction probably due to lidocaine injection (n = 1), resulting in nitrous oxide being discontinued.

## 4. Discussion

The use of nitrous oxide in pediatrics is well known and widespread [2]. However, its use is still too often limited to doctors or nurses. Other health professionals such as medical assistants are rarely included [16,25]. Indeed, various studies and guidelines indicate that the administration of nitrous oxide can be carried out by paramedical staff such as physiotherapists, dental assistants, and others. To improve pain management in units where nursing staff are not present, we aimed to assess the feasibility of administration by medical assistants who have received specific training [9,10].

The aim of our study was to evaluate the safety of medical assistants administering nitrous oxide during painful procedures in outpatient clinics. By integrating medical assistants, it was assumed that nitrous oxide would be more established and pain management improved allowing equitable access to procedural pain relief.

Non-anesthetists have been found to be able to safely administer nitrous oxide to adults and children outside operating room and similar anesthesia facilities, provided they have been trained, in many European countries and worldwide [9,11,26,27]. In Switzerland, nitrous oxide is used more and more often in pediatric wards as has been reported by a hospital in the French-speaking part of Switzerland [28]. Nitrous oxide has the advantage of being easy to use, effective within a limited time period and associated with minimal side effects. In fact, nitrous oxide does not require fasting, which makes it easy to use in procedures related to pediatric outpatient surgery [3,4,14,19]. Because of its anxiolytic action, nitrous oxide eases the discomfort of painful procedures, thereby improving the quality of care by preventing vicious circles where anxious children becoming increasingly frightened, making the procedure more difficult [9,21]. Sedation with nitrous oxide helps nursing staff because the patient is relaxed and has an altered perception of time [9]. The use of Midazolam (or chloral hydrate) is an alternative for anxious children, but it has no analgesic effect. In addition, it is slow acting and long lasting, with the risk of paradoxical effects [18]. The use of more potent drugs such as morphine or nalbuphine requires specialized staff and increased long-term monitoring.

The use of nitrous oxide alone may not be sufficient for some painful procedures. Therefore, the simultaneous use of local anesthetics with EMLA cream or lidocaine injections is recommended to achieve a better analgesic effect [6,13,14,15,29]. In our study, local anesthetics were used in more than half of the cases, with an additional analgesic in 7% of the cases (n = 23).

For the most part, there were no side effects, and the six adverse events reported were similar to those observed in other studies [5,12,15,16]. No respiratory and/or cardiovascular problems were noted, and no serious adverse events occurred. This demonstrates that the use of nitrous oxide administered by medical assistants is safe.

Pain was one of the adverse effects encountered in our study and in the two cases of pain; the procedure was stopped and carried out with other analgesics or anesthetic agents. The effectiveness of nitrous oxide can be evaluated by pain assessments but also by using distress/anxiety/fear scores. Different scales to assess these symptoms exist [30,31]. A limitation of our study is the assessment of pain. Only in two cases were validated scales (i.e., the face scale) used to determine the amount of pain. In all other situations, pain was determined based on answers of “absent”, “moderate”, or “severe”. In order to improve the use of nitrous oxide and better determine its effects, it is recommended to employ validated and reliable assessments and train staff accordingly. Fear also constitutes an important symptom, as both pain and fear are interacting emotional reactions, which are often difficult to distinguish [30]. A fear assessment scale has been developed by Thurillet et al. (2022) [30], which can be combined with a pain assessment scale. Thus, both emotions could be determined.

The need for two medical assistants for each procedure using nitrous oxide can constitute a barrier, as more resources are needed. However, the child being calmer and more relaxed certainly allows the painful procedure to be carried out in better conditions and, therefore, more quickly.

Another limitation of this study was the lack of measurement of caregiver and parent satisfaction using validated tools. Medical assistants and physicians reported high satisfaction with the use of nitrous oxide, as the advantages of this approach for the child and his parents were its good acceptance and adequate pain control. However, no specific questionnaire was completed. In order to improve the reporting, pain assessments involving a face scale, for example, should be integrated directly into the nitrous oxide assessment form, and training in the use of specific scales should be provided.

## 5. Conclusions

This study suggests that the use of nitrous oxide in the outpatient setting by trained medical assistants is feasible and effective within the specific context of this study. The introduction of nitrous oxide in the pediatric outpatient clinic has resulted in improved pain management with a high level of satisfaction from patients, parents and health professionals. This study shows that we are moving in the right direction to improve pain management during pediatric procedures performed in the outpatient setting. Further research to evaluate the pain associated with the patient, including parents and health personnel, is necessary to more precisely assess the benefits of this procedure with the implementation of validated scales. While this study provides preliminary evidence that the use of nitrous oxide by medical assistants after appropriate training might be safe, feasible, and effective, further research is necessary to establish its broader applicability.

## Figures and Tables

**Table 1:** Patient characteristics (n = 324).

					n	%
	Age (median 10 yr. (± 3.7); range 3yr. to 19 yr. old)					
	<4 yr. old				3	0.9
	4 to <8 yr. old				82	25.3
	8 to <12 yr. old				110	34
	12 to <16 yr. old				100	30.9
	≥16 yr. old				29	9
Gender						
	Female				120	37
	Male				204	63

**Table 2:** Type of procedures performed with nitrous oxide.

Procedures	n	(%)
Bone fracture reduction and cast	74	(22.8)
Onychectomy	51	(15.7)
Excision (wart, moluscum, other)	34	(10.5)
Needle puncture	26	(8.0)
Adhesiolysis	24	(7.4)
Infiltration	24	(7.4)
Burn/wound debridement	18	(5.6)
Removal of Kirschner wires	16	(4.9)
Pilonidal cyst treatment	15	(4.6)
Dressing	12	(3.7)
Cyst excision	9	(2.8)
Removal of suture threads	7	(2.2)
Abscess drainage	3	(0.9)
Foreign body removal	3	(0.9)
Skin biopsy	2	(0.6)
Laser epilation	2	(0.6)
Painful elbow pronation reduction	2	(0.6)
Closure laceration	2	(0.6)
Total	324	(100)

**Table 3:** Procedures performed using nitrous oxide with another analgesia.

Local Anesthesia Lidocaine (n = 92)	n	(%)	EMLA Cream (n = 101)	n	(%)	Systemic Analgesia (n = 23)	n	(%)
Onychectomy	44	(47.8)	Onychectomy	43	(42.6)	Bone fracture reduction and cast	17	(73.9)
Bone fracture reduction and cast	20	(21.7)	Adhesiolysis	21	(20.8)	Infiltration	3	(13.0)
Excision (wart, moluscum, other)	13	(14.1)	Excision (wart, moluscum, other)	19	(18.8)	Excision (wart, moluscum, other)	2	(8.7)
Abscess drainage	6	(6.5)	Needle puncture	18	(17.8)	Onychectomy	1	(4.3)
Infiltration	5	(5.4)						
Foreign body removal	1	(1.1)						
Skin biopsy	1	(1.1)						
Pilonidal cyst treatment	1	(1.1)						
Dressing	1	(1.1)						
Total	92	(28.4)		101	(31.2)		23	(7.1)

## Data Availability

The raw data supporting the conclusions of this article will be made available by the authors on request.
